# Determinants of subclinical leprosy among household contacts in Indonesia: serological and socio-demographic factors

**DOI:** 10.7717/peerj.20631

**Published:** 2026-01-12

**Authors:** Khariri Khariri, Sunarno Sunarno, Novaria Sari Dewi Panjaitan, Putu Yuliandari, Sarwo Handayani, Rita Marleta Dewi, Nastiti Intan Permata Sari, Fitriana Fitriana, Agriani Dini Pasiana, Ina Kusrini, Edwin Nugroho Njoto

**Affiliations:** 1 Center for Biomedical Research, Research Organization for Health, National Research and Innovation Agency (BRIN), Cibinong, West Java, Indonesia; 2 Directorate of Evaluation Policy Research and Innovation, National Research and Innovation Agency (BRIN), Cibinong, West Java, Indonesia; 3 Faculty of Medicine and Health, Institut Teknologi Sepuluh Nopember, Surabaya, East Java, Indonesia

**Keywords:** Subclinical leprosy, *Mycobacterium leprae*, BCG vaccination, Household contacts, Seroepidemiology, Indonesia, Preventive medicine

## Abstract

**Background:**

Leprosy remains a public health challenge in Indonesia, which ranks third globally after India and Brazil. Subclinical infection among household contacts contributes to ongoing transmission, as individuals infected with *Mycobacterium leprae* (*M. leprae*) without symptoms may serve as undetected reservoirs. This study investigated serological and sociodemographic determinants associated with subclinical *M. leprae* infection among household contacts of leprosy patients in Tangerang, Indonesia.

**Methods:**

A cross-sectional study was conducted in 2020 among 320 household contacts of confirmed leprosy index cases recruited through purposive sampling. Anti-Phenolic Glycolipid-1 (PGL-1) IgM antibodies were detected using an in-house enzyme-linked immunosorbent assay (ELISA). Bivariate analysis using Chi-square and *t*-tests assessed preliminary associations, and multivariate logistic regression was applied to identify independent predictors of seropositivity, adjusting for potential confounders.

**Results:**

Overall, 43.8% of household contacts were seropositive for anti-PGL-1 IgM antibodies. Multivariate analysis revealed that a history of Bacille Calmette-Guerin (BCG) vaccination was associated with significantly lower odds of seropositivity (adjusted OR = 0.514; 95% CI [0.291–0.907]; *p* = 0.018), while the presence of a visible BCG scar was associated with nearly twofold higher odds (adjusted OR = 1.953; 95% CI [1.117–3.415]; *p* = 0.024). No significant associations were found between sociodemographic factors such as age, sex, or contact duration, and seropositivity.

**Conclusion:**

BCG vaccination status and visible BCG scars were key determinants of anti-PGL-1 seropositivity, suggesting complex interactions between vaccination, immune response, and exposure to *M. leprae*. The study highlights the protective role of BCG-induced immunity while emphasizing the need for standardized scar assessment and continuous surveillance of household contacts. Although limited by its cross-sectional and purposive design, the integration of immunological and epidemiological data represents a strength, providing evidence to support Indonesia’s Zero Leprosy 2030 control strategy.

## Introduction

Leprosy, or Hansen’s disease, is a chronic infectious disease caused by *Mycobacterium leprae* (*M. leprae*) that primarily affects the skin and peripheral nerves. Although the global incidence of leprosy has decreased from more than five million cases in the 1980s to 133,802 cases in 2021, new cases continue to be reported, especially in endemic areas (World Health Organization (WHO), 2021, 2022). Indonesia successfully eliminated leprosy by reducing its prevalence to less than one case per 10,000 individuals in 2000. This achievement led to a significant reduction in the national leprosy control program in Indonesia (World Health Organization (WHO), 2010). However, in recent years, the number of reported cases has increased, with 12,000–17,000 new cases reported annually. This rise has been exacerbated by the Corona Virus Disease 2019 (COVID-19) pandemic, resulting in an incidence exceeding four cases per 100,000 population, placing Indonesia as the third highest in leprosy incidence after India and Brazil (Sebong et al., 2025). The subclinical form of leprosy is concerning because individuals infected with *M. leprae* but not exhibiting clinical symptoms can potentially be sources of transmission, thereby increasing the risk of spread within communities (Sugawara-Mikami et al., 2022). Therefore, identifying the determinants of subclinical leprosy is crucial for its control and prevention. A systematic review by deAlecrin et al. (2022) found an association between illness and the absence of Bacille Calmette-Guerin (BCG) scars, with several studies identifying that the presence of a BCG vaccine scar was considered a protective factor against developing leprosy. The BCG vaccine has been proven to provide protective effects against leprosy at varying levels, despite initially being developed for tuberculosis (Coppola et al., 2018).

Serological factors have been extensively studied for the detection of subclinical *M. leprae* infections. The detection of specific antibodies, such as Anti-Phenolic Glycolipid-1 (PGL-1) immunoglobulin M (IgM), has been used as a biomarker to identify individuals at a higher risk of developing clinical leprosy (Pierneef et al., 2024). Previous studies have shown that high levels of anti-PGL-1 antibodies correlate with exposure to *M. leprae* and the likelihood of disease progression (Penna et al., 2016). However, the sensitivity and specificity of serological methods for detecting subclinical leprosy remain challenging, necessitating a multifaceted approach to assess the risk among household contacts.

In addition to serological factors, sociodemographic factors also play a significant role in the risk of *M. leprae* infection. Age, sex, economic status, housing conditions, and population density have been associated with an increased risk of leprosy, including its subclinical forms (Feenstra et al., 2013). Household contact with leprosy index cases, especially those with close family relationships, increases the likelihood of continuous exposure to *M. leprae*, which can enhance the chances of infection (Sales et al., 2011). Therefore, analyzing socio-demographic factors is crucial for understanding the spread of subclinical leprosy and developing community-based intervention strategies.

A risk-based approach to controlling subclinical leprosy requires the integration of serological testing and evaluation of socio-demographic factors. Understanding the relationship between immune responses and the characteristics of exposed populations can enhance early detection efforts, allowing for more effective interventions before the disease progresses to more severe clinical forms (Bathula, Khurana & Singh, 2023). Serological surveillance programs, along with social epidemiological approaches, can aid in identifying individuals at high risk for earlier medical intervention.

Understanding subclinical infection among household contacts who represent the population at highest risk of developing active disease is critical for strengthening Indonesia’s national leprosy control efforts. In alignment with the Zero Leprosy 2030 strategy, this study aimed to identify the determinants of subclinical leprosy by focusing on both serological and sociodemographic factors among household contacts of confirmed leprosy index cases. By integrating immunological and epidemiological approaches, the findings are expected to contribute to early detection strategies, targeted surveillance, and preventive interventions that support public health policies in controlling *M. leprae* transmission in endemic communities across Indonesia.

### Study design

This study employed a cross-sectional design with an observational, laboratory-based, descriptive approach.

### Time and location

The study was conducted in 2020 at Dr. Sitanala Hospital and two affiliated primary healthcare centers, the Neglasari and Kedaung Wetan Community Health Centers, located in Tangerang, Banten Province. These facilities were selected because Dr. Sitanala Hospital serves as a national referral and rehabilitation center for leprosy in Indonesia and maintains comprehensive medical records of confirmed cases. The Neglasari and Kedaung Wetan health centers manage surrounding endemic communities that actively participate in the national contact surveillance and case detection programs. Data from confirmed leprosy index cases and their household contacts were obtained from medical records collected during 2015–2019 at these facilities, which were then used to identify and determine eligible sampling locations for this study. In 2020, household visits were conducted for sample collection involving family members living with leprosy index cases who were undergoing or had recently completed treatment at the designated sites. Blood specimen collection was performed at the residences of household contacts, and laboratory examinations were conducted by the Leprosy Laboratory, Institute of Tropical Disease (ITD), Universitas Airlangga.

### Population and samples selection

The study population comprised families with members who had undergone leprosy treatment at Dr. Sitanala Hospital, the Neglasari Community Health Center, and the Kedaung Wetan Community Health Center. The study sample included household contacts, defined as family members living with leprosy index cases receiving treatment at these health facilities. One to two household contacts were recruited from each index case and selected using purposive sampling based on predefined inclusion and exclusion criteria among household contacts of confirmed leprosy index cases (Etikan, 2016). The study included household members of leprosy index cases undergoing treatment who exhibited no clinical signs of leprosy, were aged ≥1 year, and provided written informed consent (with parental or guardian consent for minors). Household contacts were excluded if they had severe illness, a history of bleeding disorders such as hemophilia or idiopathic thrombocytopenic purpura (ITP), chronic illnesses requiring regular anticoagulant medication, or were diagnosed with leprosy or tuberculosis by a healthcare professional. Individuals with other medical conditions that prevented blood sampling, as determined by a physician, were also excluded. This study was conducted with ethical clearance No. LB.02.01/2/KE.164/2019.

### Serological assay

Serological testing to detect anti-PGL-1 IgM antibodies was conducted by the Leprosy Laboratory, Institute of Tropical Disease (ITD), Universitas Airlangga, using an in-house enzyme-linked immunosorbent assay (ELISA) method. The laboratory provided anti-PGL-1 IgM titer values, which were further classified as seropositive or seronegative based on internally defined cut-off values. Seronegativity was classified as anti-PGL-1 IgM <605 μ/mL, while seropositivity was classified as anti-PGL-1 IgM ≥605 μ/mL (Astari et al., 2021; Srihartati & Agusni, 2010).

### BCG vaccination status and scar assessment

BCG vaccination status and the presence of a BCG scar were treated as independent variables. Vaccination history was obtained through self-report during structured interviews, while visible BCG scars were confirmed through direct physical examination by trained field staff. This distinction was made because not all individuals with confirmed vaccination retain visible scars, and conversely, some scars may be misclassified due to unrelated skin lesions. The majority of participants received BCG during infancy as part of Indonesia’s Expanded Programme on Immunization; however, precise vaccination dates or medical records were unavailable. Both variables were therefore analyzed separately to account for differences between recalled vaccination history and observable physical evidence of prior immunization.

### Data collection

Before conducting the interviews, trained personnel explained the purpose and objectives of the study in simple and understandable language. Parental or guardian consent was obtained for minors through a signed Informed Consent Form (ICF), which is provided in File S1. A structured questionnaire was used to gather information on the factors influencing disease transmission. Following the interview, blood samples were collected and subjected to serological testing using *in-house* ELISA performed by the Leprosy Laboratory, Institute of Tropical Disease (ITD), Universitas Airlangga.

### Data management and analysis

The collected data were systematically processed, cleaned, and analyzed using the IBM SPSS Statistics version 25. Data management included checking for completeness, handling missing values, and ensuring data accuracy prior to statistical analysis. Bivariate and multivariate approaches were employed. Bivariate analysis was performed to assess the differences or associations between the independent and dependent variables. The Chi-square test was used to evaluate associations between categorical variables, while the *t*-test was applied to compare the mean or median values of continuous variables between the two groups, depending on the data distribution. Variables with a *p*-value < 0.25 in the bivariate analysis or those considered biologically relevant based on previous literature were included in the multivariate logistic regression model to control for potential confounding effects and to identify independent predictors. Multivariate logistic regression analysis was applied to assess the strength of the associations between multiple independent variables and the outcome variable, adjusting for potential confounding factors. Logistic regression was applied to estimate odds ratios (OR) and 95% confidence intervals (CI), representing the likelihood of anti-PGL-1 IgM seropositivity among exposed compared to unexposed groups. This approach is appropriate for cross-sectional data with binary outcomes, where ORs serve as measures of association rather than causality. All statistical tests were two-tailed, and a *p*-value of <0.05 was considered statistically significant.

## Results

**Figure 1:**
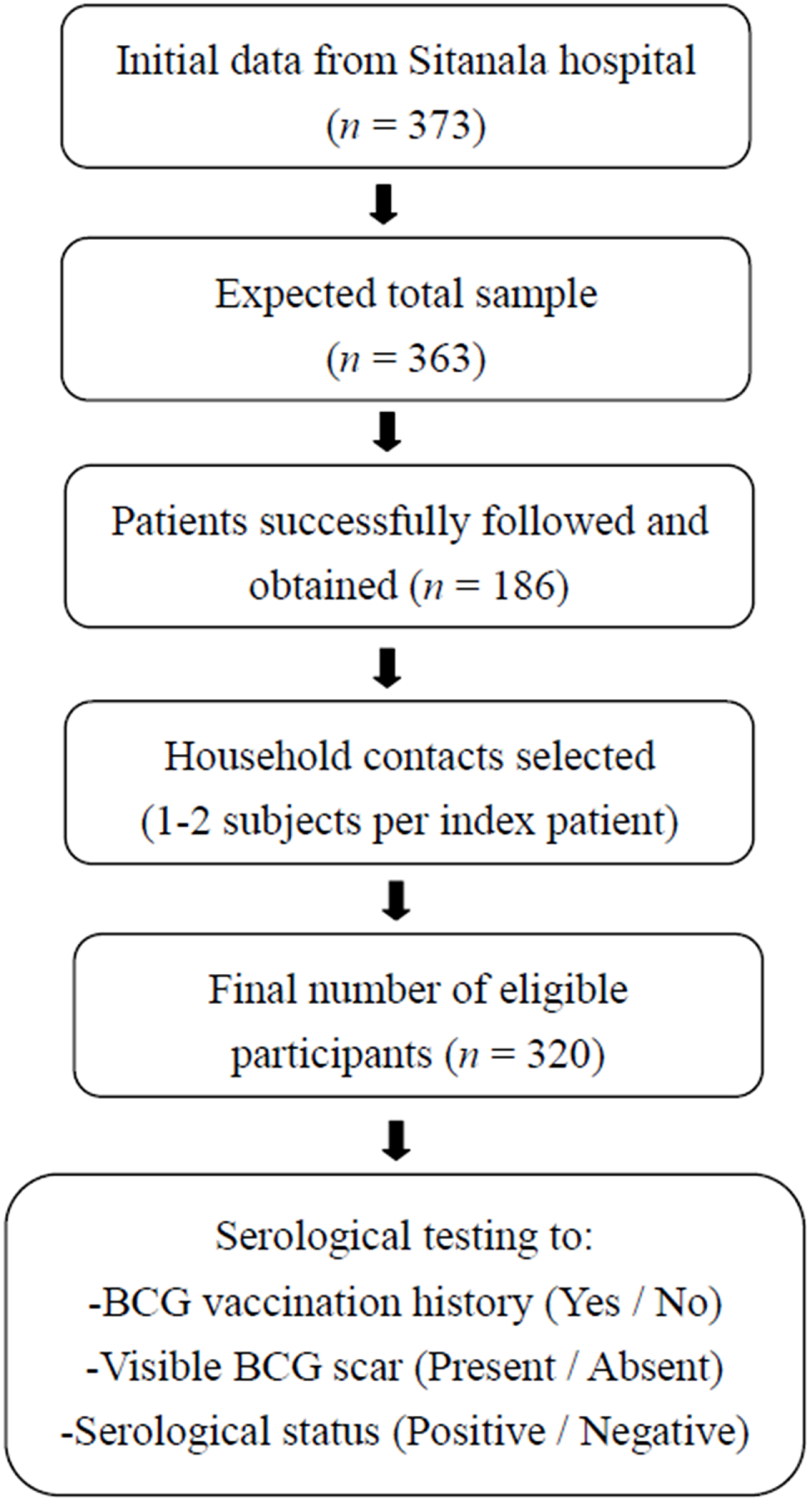
Recruitment of household contacts from leprosy index case. The flowchart illustrates the stepwise process used to recruit household contacts of leprosy index cases for inclusion in this study. Initial data were obtained from 373 registered patients with leprosy at Dr. Sitanala Hospital. From these, 186 index cases were successfully located and followed up, and one to two household contacts were selected from each index case. A total of 320 household contacts met the eligibility criteria and were enrolled in the study. Furthermore, all household contacts underwent anti-PGL-1 IgM serological testing and were categorized based on their BCG vaccination history, visible BCG scar, and antibody status.

The initial data were obtained from the patient list at Dr. Sitanala Hospital, which recorded a total of 373 patients. However, not all patients on the list could be contacted. Only half of the residents could be visited. One to two subjects were selected as household contacts from each index case. Based on the selection process and residence visits, 320 household contacts were included in this study (Fig. 1).

**Figure 2:**
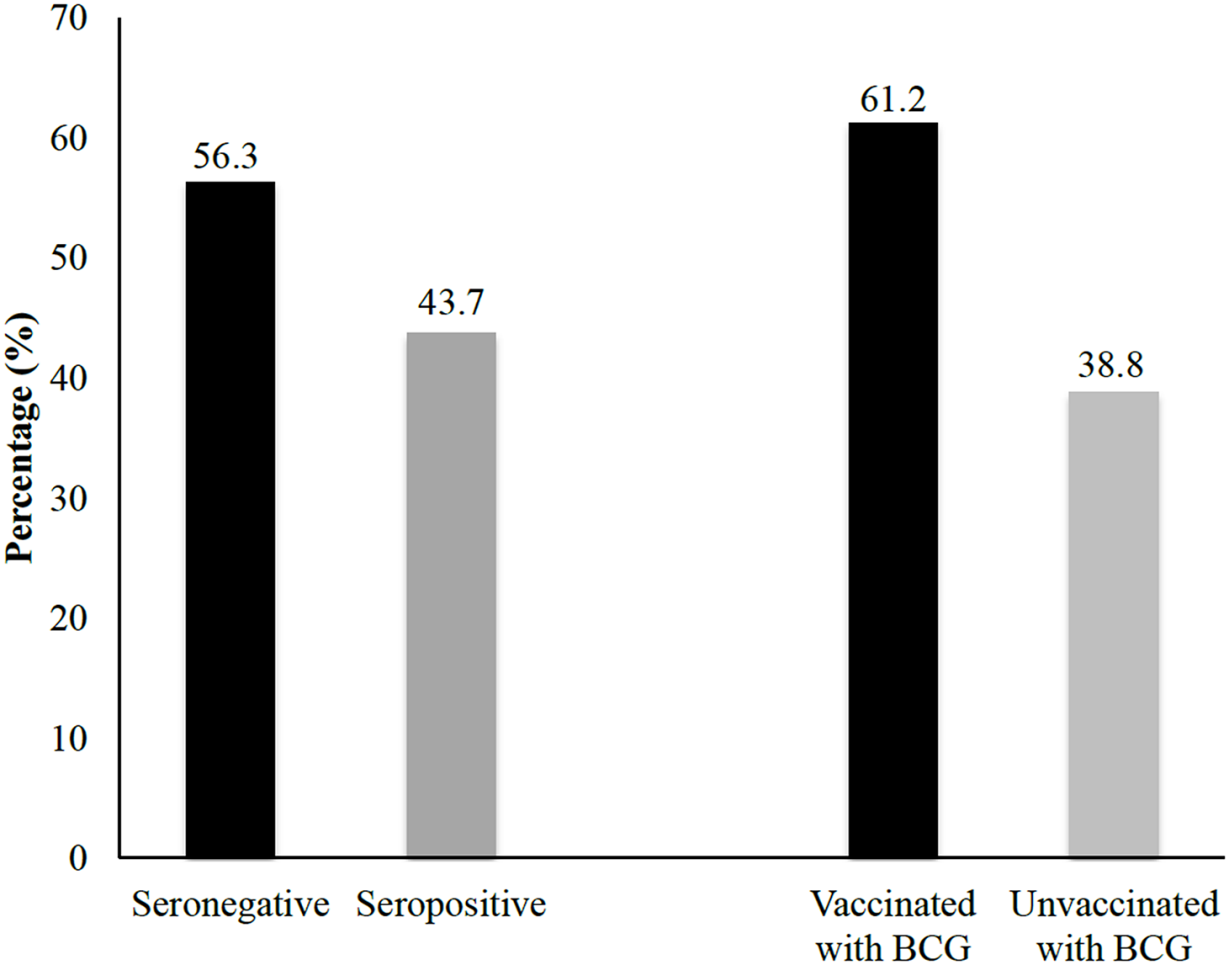
Univariate analysis of seropositivity based on BCG vaccination status. The percentages of seropositivity and seronegativity among household contacts with and without a history of BCG vaccination are shown.

This study investigated the determinant factors influencing seropositive antibody prevalence against *M. leprae* using bivariate and multivariate analyses. The findings indicated several environmental and sociodemographic variables that might influence seropositivity, although the statistical significance differed depending on the parameter. Figure 2 illustrates the distribution of serological and vaccination status among household contacts. Laboratory results indicated that approximately 43.8% of household contacts tested seropositive, suggesting prior exposure to the pathogen or the presence of immune response markers. In contrast, 56.3% of household contacts were classified as seronegative, indicating no detectable antibodies at the time of testing. Additionally, the data revealed that 61.3% of household contacts had been vaccinated in the past, signifying a substantial proportion of individuals with induced immunity through immunization programs. In contrast, 38.8% of household contacts had not been vaccinated.

Table 1 provides a detailed summary of the laboratory results categorized according to various demographic characteristics. The proportions of seropositive and seronegative individuals were studied across multiple parameters, including sex, age group, marital status, education level, occupation, income, contact length, room density, vaccination status, presence of scar tissue, personal hygiene, and nutritional status. The findings indicated that more than 40% of household contacts tested seropositive in both male and female groups, with no statistically significant differences observed between the sexes (*p* = 0.795). Similarly, no notable variations in seropositivity were detected across the different age groups (*p* = 0.632). However, the highest proportion of seropositive individuals was observed in the 35–44 age range, followed closely by those in the 25–34 age range. The corresponding raw data supporting these findings are available in File S2.

**Table 1:** Respondent characteristics and laboratory results.

Characteristics		Seronegative		Seropositive		Total	
		*n*	%	*n*	%	*n*	Sig.
Sex	Male	72	57.10%	54	42.90%	126	0.795
	Female	108	55.70%	86	44.30%	194	
Age group	1–4	9	69.20%	4	30.80%	13	0.632
	5–9	22	55.00%	18	45.00%	40	
	10–14	30	54.50%	25	45.50%	55	
	15–24	52	55.90%	41	44.10%	93	
	25–34	19	51.40%	18	48.60%	37	
	35–44	20	51.30%	19	48.70%	39	
	45–54	15	71.40%	6	28.60%	21	
	55–64	10	52.60%	9	47.40%	19	
	65–74	3	100.00%	0	0.00%	3	
Marital status	Not married yet	116	57.70%	85	42.30%	201	0.31
	Married	56	51.40%	53	48.60%	109	
	Divorced	8	80.00%	2	20.00%	10	
Education level	No education	60	61.20%	38	38.70%	98	0.761
	Elementary school	34	51.50%	32	48.50%	66	
	Junior high school	33	53.20%	29	46.80%	62	
	Senior high school	52	55.90%	41	44.10%	93	
	Bachelor degree	1	100.00%	0	0.00%	1	
Occupation	Un-employed	135	55.10%	110	44.90%	245	0.347
	Employed	45	59.20%	31	40.70%	76	0.545
Family member status	Husband/wife	33	56.9%	25	43.1%	58	
	Child	89	56.0%	70	44.0%	159	
	Grandchild	34	59.6%	23	40.4%	57	
	Daughter/son-in-law	5	33.3%	10	66.7%	15	
	Parents	8	66.7%	4	33.3%	12	
	Other	11	57.9%	8	42.1%	19	
Income	High	28	56.00%	22	44.00%	50	0.969
	Low	152	56.30%	118	43.70%	270	
Contact- length	≦2 years	5	83.30%	1	16.70%	6	0.177
	>2 years	175	55.70%	139	44.30%	314	
Room density	<8 m^2^/2 persons	50	61.00%	32	39.00%	82	0.31
	≧8 m^2^/2 persons	130	54.60%	108	45.40%	238	
Hygiene	Good	169	56.50%	130	43.50%	299	0.71
	Less	11	52.40%	10	47.50%	21	
BCG vaccine	Yes	105	53.60%	91	46.40%	196	0.22
	No	75	60.50%	49	39.50%	124	
Scar tissue	Yes	112	59.30%	77	40.70%	189	0.19
	No	68	51.90%	63	48.10%	131	

In the univariate analysis, none of the evaluated factors, including contact length (*p* = 0.177), BCG vaccination history (*p* = 0.22), and presence of a visible scar (*p* = 0.19), were statistically associated with seropositivity (*p* < 0.05). However, multivariate analysis was still conducted to assess the potential confounding variables and identify independent predictors that may have borderline significance or biological relevance. Therefore, three variables (BCG vaccination history, visible BCG scar, and contact length with the leprosy index cases) with *p* < 0.25 were further analyzed. In the multivariate model, both BCG vaccination history (*p* = 0.018) and visible BCG scars (*p* = 0.024) emerged as independent predictors of seropositivity. In contrast, contact length (*p* = 0.142) did not show a significant association.

Our bivariate analysis also revealed that factors such as marital status (*p* = 0.31), level of education (*p* = 0.761), type of occupation (*p* = 0.347), and income level (*p* = 0.969) do not appear to influence the leprae seropositivity in a statistically significant manner. Likewise, household conditions, including room density (*p* = 0.31) and personal hygiene practices (*p* = 0.71), did not show a discernible impact on the presence of antibodies. Individuals with contact length exceeding two years had a higher seropositivity rate (44.3%) than those with shorter contact (<2 years, 16.7%). Although this difference was not statistically significant (*p* = 0.177), contact length was a potential variable in previous studies; therefore, it was included in the multivariate analysis (Ortuno-Gutierrez et al., 2019; Ronse et al., 2022). Moreover, vaccination status appears to play a crucial role in the seropositivity rates. Household contacts who received the BCG vaccine were less likely to test seropositive than those who remained unvaccinated, although this difference was not statistically significant (*p* = 0.22). Additionally, individuals with visible scar tissue from a previous vaccination were less likely to be seropositive than those without such markings (*p* = 0.19).

Table 2 shows the multivariate analysis of several factors associated with seropositivity based on laboratory results, highlighting the key determinants that may influence immune response patterns within the population. To ensure a more accurate interpretation of the relationships identified, the distinction between unadjusted (bivariate) and adjusted (multivariate) analyses is clarified below, emphasizing the independent predictors that remained statistically significant after controlling for potential confounders. It is important to note that while several variables showed non-significant associations in the bivariate analysis (*p* > 0.05), multivariate logistic regression identified two factors that remained statistically significant after adjustment for potential confounders: BCG vaccination history and the presence of a visible BCG scar. Specifically, individuals who reported having received BCG vaccination had significantly lower odds of anti-PGL-1 IgM seropositivity (adjusted OR = 0.514; 95% CI [0.291–0.907]; *p* = 0.018), whereas those with a visible BCG scar demonstrated nearly a twofold higher likelihood of being seropositive (adjusted OR = 1.953; 95% CI [1.117–3.415]; *p* = 0.024). These findings indicate that vaccine-related variables were independent predictors of antibody response, even after controlling for other demographic and environmental factors. In contrast, contact duration, although biologically relevant, did not reach statistical significance in the adjusted model (*p* = 0.142).

**Table 2:** Multivariate analysis of factors associated with seropositivity.

	Sig.	Crude OR	Sig	Adjusted OR	95% CI for adjusted OR	
					Lower	Upper
BCG vaccine a	0.018	0.504	0.022	0.514	0.291	0.907
Tissue Scar a	0.024	1.904	0.019	1.953	1.117	3.415
Contact-length a	0.214	3.958				
	0.142	0.200	0.077	0.761		

**Note:**

a. Variable(s) entered on step 1: vaccine, scar tissue, contact length.

## Discussion

Subclinical leprosy, characterized by the presence of *M. leprae* without noticeable symptoms, is a concern because of its potential for transmission. Serological assays are commonly used to detect subclinical leprosy seropositivity by detecting the presence of antibodies against *M. leprae* antigens. Our study used the enzyme-linked immunosorbent assay (ELISA) IgM antibody of phenolic glycolipid-1 (PGL-1) titer for seropositivity testing. A systematic review by Penna et al. (2016) found a clear and consistent link between anti-PGL-1 positivity and the development of leprosy in healthy contacts (Penna et al., 2016). Although the etiology of leprosy has been discovered and the disease is curable, there is a lack of efficient diagnostic tools and treatment strategies. The stigma associated with social and cultural prejudices remains a concern. The combination of these factors makes it difficult to identify at-risk populations early on, which is why disease control efforts should prioritize protecting these people to stop the disease and decrease social and physical disabilities (Santacroce et al., 2021).

In this study, we analyzed the significance and relationship of sex, age, marital status, educational level, occupation, marital status, income, contact length to the leprosy index case, room density (either <8 m^2^/2 persons or ≧8 m^2^/2 persons), hygiene, BCG vaccine, and scar tissue to do BCG vaccination, to the observed leprosy seropositivity. A systematic review by Pertiwi & Syahrul (2024) found that the key risk factors for leprosy include household density and personal hygiene, while a history of BCG vaccination serves as a protective factor. However, our multivariate analysis showed that only BCG vaccination and the presence of scar tissue were significant factors. The divergent associations observed between BCG vaccination and visible scar presence may reflect biological and observational differences. While vaccination history likely represents true immunization exposure, visible scarring can be influenced by host-specific healing responses, the technique of vaccine administration, or scar misclassification. Therefore, these two indicators should not be interpreted interchangeably but rather as complementary proxies for prior vaccination and variability in immune response.

The clinical characteristics of index cases play a critical role in the infection risk among contacts. Most *M. leprae* infections occur in untreated multibacillary (MB) individuals. The relative risk of contracting leprosy is estimated to be five to ten times higher in household contacts of MB index cases than in the general community (Hambridge et al., 2021). However, data on the clinical status of leprosy index cases in our study, whether paucibacillary or multibacillary, were not available. Although contact length has been consistently highlighted as an important risk factor for leprosy transmission in previous studies, Fine et al. (1997) and Moet et al. (2006) showed that prolonged close contact with multibacillary patients substantially increased the risk of developing the disease (Fine et al., 1997; Moet et al., 2006). Our study did not find contact duration to be a statistically significant predictor of seropositivity. Household contacts with more than two years of exposure to leprosy patients did not show significantly higher seropositivity rates than those with shorter contact durations. One possible explanation is that most index cases in our sample were already receiving treatment, which reduces infectivity and thereby attenuates the impact of prolonged exposure. A study by Niitsuma et al. (2025) showed that there were unmeasured confounders that could be related to the contact length effect, such as heterogeneity in immune responses, intensity of exposure, or genetic susceptibility among household members (Niitsuma et al., 2025).

Another important consideration is the timing of sample collection among household contacts relative to the treatment completion of the index cases. Because this study utilized retrospective data from 2015–2019 and adopted a cross-sectional design, precise intervals between the end of treatment in index cases and the timing of blood collection for ELISA could not be established. Most household contacts were visited after the index cases had finished multidrug therapy, at which point bacillary load and infectivity are generally minimal. Therefore, the detected anti-PGL-1 IgM antibodies in our cohort are more likely to represent cumulative or historical exposure rather than recent transmission. This temporal aspect may partly explain why contact duration was not significantly associated with seropositivity in our analysis. Future longitudinal studies incorporating precise temporal mapping of exposure and sampling are warranted to better elucidate the kinetics of anti-PGL-1 antibody responses and their potential role in identifying recent *M. leprae* transmission.

The BCG vaccine has been proven to provide protective effects against leprosy at varying levels, despite initially being developed for tuberculosis (Coppola et al., 2018). The protective effect of the BCG vaccine was demonstrated in a meta-analysis by Pertiwi & Syahrul (2024), which estimated a protective efficacy ranging from 23% (95% CI [14–37%]) in experimental studies to 61% (95% CI [51–70%]) in observational studies. In our statistical analysis, BCG vaccination was significantly associated with reduced seropositivity in the multivariate model (*p* = 0.018, adjusted OR = 0.514, 95% CI [0.291–0.907]). This finding highlights the importance of BCG-related immunity, whereas the impact of contact duration may be influenced by other unmeasured factors, such as proximity or intensity of exposure.

Several previous studies have used case-control and cohort methods to test the efficiency of the BCG vaccine against leprosy and tuberculosis, using the existence of a BCG scar as a sign of prior BCG vaccination (Sterne et al., 1996). Interestingly, based on our multivariate analysis, we found that the scar due to BCG vaccination was significantly associated with leprosy seropositivity, with almost double the possibility (Sig. 0.019, adjusted OR = 1.953). The presence of a BCG scar was used as a proxy indicator, which may not fully reflect the recent or repeated vaccination status. While a documented history of vaccination generally indicates exposure to live attenuated *M. bovis* BCG and associated protective immunity, scar formation is a variable dermatologic outcome that depends on factors such as vaccine strain, injection technique, host skin reactivity, and healing response. A previous study by Zodpey et al. (2007) reported that neither leprosy nor tuberculosis protective immunity conferred by vaccination was associated with a larger BCG scar size. Some individuals who develop more intense local reactions may produce both a visible scar and stronger antibody responses, which could explain the higher seropositivity observed among those with scars. Conversely, individuals who were vaccinated but no longer display a visible scar may still retain systemic immune protection, reflected in lower seropositivity rates. This discrepancy underscores the need for standardized assessment of BCG scarring and immunological profiling in future studies to better interpret serological patterns in leprosy-endemic settings.

A notable strength of this study is its integration of immunological and epidemiological data to explore subclinical infection within a real-world endemic setting. The use of a validated in-house ELISA method, standardized across all samples, ensured reliable antibody detection. Furthermore, this study provides seroepidemiological data from Indonesia, which remains underrepresented in regional leprosy research. The inclusion of household contacts across multiple healthcare centers also enhances the representativeness of the findings for community-based transmission settings.

The limitation of this study is the lack of categorical seropositivity evaluation, which was intended to distinguish strong, moderate, and weak antibody responses but was not performed due to an uncertain internal cut-off. A qualitative interpretation of the ELISA results (seropositive/seronegative) was therefore considered sufficient to address the research question regarding exposure and risk determinants. Another limitation is the absence of precise data regarding the interval between the completion of treatment in index cases and blood sampling among household contacts, which may have influenced the observed antibody responses and limited interpretation of recent *vs* past exposure. Furthermore, as this study employed a cross-sectional design, the observed associations between BCG vaccination status, visible scar presence, and anti-PGL-1 IgM seropositivity should be interpreted as correlational rather than causal. Although multivariate analysis was used to control for several potential confounders, unmeasured variables such as genetic susceptibility, intensity of contact exposure, or other immunological factors may have influenced the outcomes. This residual confounding is an inherent limitation of observational studies and may have contributed to the variability in serological responses observed among household contacts.

As this study employed purposive non-probability sampling, the findings are representative only of the sampled population and cannot be generalized to all household contacts in Indonesia. However, the use of inferential statistics remains valid for identifying associations within this defined analytical sample, provided that conclusions are interpreted within this contextual limitation.

In addition, the discordant relationship observed between BCG scar presence and seropositivity may result from variability in scar persistence, misclassification during examination, or individual differences in immune reactivity; this finding warrants further investigation in longitudinal and immunologically controlled studies. Moreover, we were unable to determine the correlation between the exact timing of BCG vaccination for each index case and leprosy, as most participants had received it in early childhood and did not retain vaccination records.

Despite these limitations, the study adds valuable evidence supporting the role of BCG vaccination in modulating humoral immune responses to *M. leprae* and underscores the need for continued surveillance of household contacts. Future studies incorporating full quantitative ELISA data together with clearly defined exposure timelines could provide more detailed insights into immune response dynamics and the risk of subclinical infection.

## Conclusion

This study identified several factors associated with subclinical leprosy among household contacts in Tangerang, Indonesia. The seroprevalence of anti-PGL-1 IgM, an indicator of *Mycobacterium leprae* exposure, was significantly influenced by BCG vaccination status and the presence of a visible BCG scar. BCG vaccination was associated with lower odds of seropositivity, suggesting a possible protective immunological effect, whereas the presence of a visible scar showed an opposite trend, indicating potential variability in individual immune responses or scar persistence. This discordance highlights the complexity of using BCG scarring as a surrogate marker of protective immunity and underscores the need for standardized evaluation of vaccination status in future studies.

Because most household contacts were sampled after the completion of treatment in index cases, the detected antibody responses likely reflect cumulative rather than recent exposure. Sociodemographic variables such as age, sex, and duration of contact showed no statistically significant association with seropositivity, possibly due to reduced infectivity following multidrug therapy. Collectively, these findings reinforce the importance of maintaining high BCG vaccination coverage and systematic contact surveillance as essential components of leprosy control. Future longitudinal studies incorporating quantitative serological data, precise exposure timelines, and detailed immunological profiling are warranted to clarify antibody kinetics and the long-term protective role of BCG-induced immunity in endemic settings.

## Supplemental Information

10.7717/peerj.20631/supp-1 Supplemental Information 1 Raw Data of Serological and Demographic Characteristics of Household Contacts. The complete dataset containing demographic variables, serological results, and other parameters analyzed in this study.

## Abbreviation

Anti-PGL-1: Anti Phenolic Glycolipid-1
IgM: Immunoglobulin M
BCG: Bacille Calmette-Guerin
OR: Odds Ratio
CI: Confidence Interval
*M. leprae*: *Mycobacterium leprae*
ELISA: Enzyme-Linked Immunosorbent Assay
COVID-19: Corona Virus Disease 2019
ITP: Idiopathic Thrombocytopenic Purpura
ICF: Informed Consent Form
ITD: Institute of Tropical Disease
MB: Multibacillary
WHO: World Health Organization

## Additional Information and Declarations

### Data Availability

The following information was supplied regarding data availability:

The raw data are available in the Supplemental File.
